# Modeling Neutralization Kinetics of HIV by Broadly Neutralizing Monoclonal Antibodies in Genital Secretions Coating the Cervicovaginal Mucosa

**DOI:** 10.1371/journal.pone.0100598

**Published:** 2014-06-26

**Authors:** Scott A. McKinley, Alex Chen, Feng Shi, Simi Wang, Peter J. Mucha, M. Gregory Forest, Samuel K. Lai

**Affiliations:** 1 Mathematics Department, University of Florida, Gainesville, Florida, United States of America; 2 Departments of Mathematics and Applied Physical Science, University of North Carolina – Chapel Hill, Chapel Hill, North Carolina, United States of America; 3 Statistical and Applied Mathematical Sciences Institute, Research Triangle Park, North Carolina, United States of America; 4 Division of Molecular Pharmaceutics, Eshelman School of Pharmacy, University of North Carolina – Chapel Hill, Chapel Hill, North Carolina, United States of America; 5 UNC/NCSU Joint Department of Biomedical Engineering, University of North Carolina – Chapel Hill, Chapel Hill, North Carolina, United States of America; Emory University School of Medicine, United States of America

## Abstract

Eliciting broadly neutralizing antibodies (bnAb) in cervicovaginal mucus (CVM) represents a promising “first line of defense” strategy to reduce vaginal HIV transmission. However, it remains unclear what levels of bnAb must be present in CVM to effectively reduce infection. We approached this complex question by modeling the dynamic tally of bnAb coverage on HIV. This analysis introduces a critical, timescale-dependent competition: to protect, bnAb must accumulate at sufficient stoichiometry to neutralize HIV *faster* than virions penetrate CVM and reach target cells. We developed a model that incorporates concentrations and diffusivities of HIV and bnAb in semen and CVM, kinetic rates for binding (k_on_) and unbinding (k_off_) of select bnAb, and physiologically relevant thicknesses of CVM and semen layers. Comprehensive model simulations lead to robust conclusions about neutralization kinetics in CVM. First, due to the limited time virions in semen need to penetrate CVM, substantially greater bnAb concentrations than *in vitro* estimates must be present in CVM to neutralize HIV. Second, the model predicts that bnAb with more rapid k_on_, almost independent of k_off_, should offer greater neutralization potency *in vivo*. These findings suggest the fastest arriving virions at target cells present the greatest likelihood of infection. It also implies the marked improvements in *in vitro* neutralization potency of many recently discovered bnAb may not translate to comparable reduction in the bnAb dose needed to confer protection against initial vaginal infections. Our modeling framework offers a valuable tool to gaining quantitative insights into the dynamics of mucosal immunity against HIV and other infectious diseases.

## Introduction

During vaginal transmission of HIV-1, virions in semen must traverse the thin layer of cervicovaginal mucus (CVM) coating the vaginal epithelium before they can encounter and potentially infect target cells (lymphocytes, macrophages, dendritic cells and Langerhans cells). Due to the presence of substantial quantities of secreted and transudated antibodies (Ab) [1], [2], CVM possesses both diffusional and immunological barrier properties against sexually transmitted viruses. In women with healthy vaginal microflora, lactobacilli secrete substantial levels of lactic acid, producing an acidic (pH ∼3.5–4) environment that inactivates leukocytes within minutes [3]. Thus, few immune cells capable of opsonization and antibody-dependent cell-mediated cytotoxicity (ADCC) are actually present in healthy CVM secretions, which also exhibit limited complement activity [4]–[6]. Neutralization, a process in which secreted or topically-applied Ab engage the gp120/gp41 trimeric glycoproteins (Env) on HIV at sufficient stoichiometry to preclude their attachment to target cells, is thus generally thought to be a critical component of sterilizing immunity against initial HIV infections in the vagina [7]. Effective neutralization in the vaginal lumen that directly reduces the rates of *acquiring* initial infections, rather than attempting to *clear* infections, may be especially important since HIV infections remain difficult to cure once established.

In response to the tremendous genetic diversity of HIV, a series of monoclonal Ab capable of broadly neutralizing diverse strains of HIV across different clades (abbreviated here as bnAb) have been recently discovered that not only neutralize a much greater diversity of HIV strains than previously, but also extend the *in vitro* geometric mean IC_50_ (the concentration necessary to reduce infectivity by 50%) into ng/mL potencies [8]–[10]. Because of the high affinity of typical Ab-antigen binding, it is generally assumed that these potent bnAb rapidly bind to and neutralize HIV. However, viruses that transmit at mucosal surfaces have generally evolved to readily penetrate mucus [11]. Indeed, HIV virions (diameter ∼100 nm) exhibit rapid diffusion in pH-neutralized CVM, enabling their rapid penetration across physiologically thick mucus layers in tens of minutes [12]. Thus, there is a very limited time window during which bnAb must accumulate on HIV at or above the neutralizing threshold before the virions can successfully diffuse across CVM and reach target cells. This challenge is not readily captured by most *in vitro* studies, where the majority of assays evaluate protection by incubating Ab with HIV for defined durations (e.g., 60 mins, some extending to overnight) prior to assaying infection of target cells over a 48–72 hr period. This procedure likely ensures greater Ab coverage on HIV prior to their exposure to target cells *in vitro*. It remains unclear whether bnAb in CVM at *in vitro* IC_50_ or IC_80_ concentrations can achieve neutralization potencies comparable to those measured *in vitro* within the limited time window before virions successfully penetrate mucus and infect target cells, or if not, how much more bnAb may be needed to achieve such sterilizing immunity in the human vagina.

To address these competing processes and their respective timescales, as well as gain insight into the dynamics of vaginal HIV infection, we developed a mathematical model that captures the competition between bnAb accumulation on the fusion-competent envelope glycoprotein of HIV and HIV penetration of CVM from semen in the lumen. Using previous measurements of HIV mobility and Ab diffusivities in human genital secretions, estimates of CVM thickness, and binding affinities for different bnAb based on surface plasmon resonance (SPR) measurements, we model the minimum Ab levels in CVM necessary to achieve 50% and 80% coverage of the HIV Env spikes before HIV virions can reach the vaginal epithelium. We also present theoretical binding affinities for bnAb that may enable protective immunity against HIV in the human vagina.

## Materials and Methods

### Defining model parameters capturing vaginal transmission of cell-free HIV

**Figure 1:**
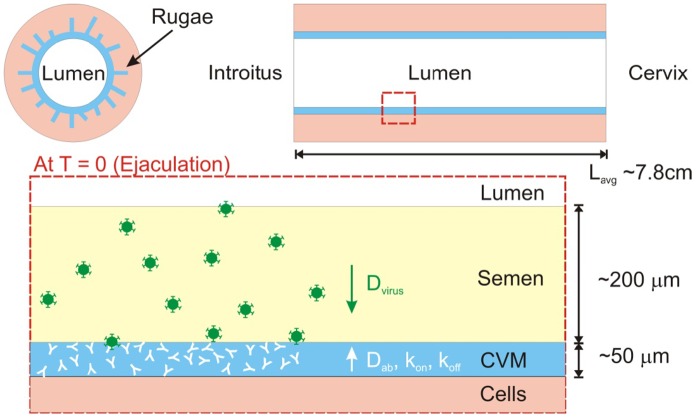
Schematic of our model for HIV diffusion from seminal secretions across antibody-laden cervicovaginal mucus (CVM) layer to underlying vaginal epithelium. To reduce infection, we assume Ab must bind to HIV before virions successfully reach the vaginal epithelium.

Our mathematical model describes the dynamics of male-to-female HIV transmission, beginning the instant semen is ejaculated into the vaginal lumen and tracking HIV virions until they reach the vaginal epithelium (see Figure 1 for schematic; Table 1 lists the various input parameters). Once virions reach the epithelial lumen, virions must still access target cells in the epithelium, and intact stratified vaginal epithelia has long been believed to serve as a mechanical barrier excluding virus access. Nevertheless, HIV virions have been observed to quickly penetrate the superficial layers of the stratified epithelium in human cervical explants and the female rhesus macaque genital tract, thereby gaining access to superficial Langerhans cells and CD4 T cells [13], [14]. The timescale required for successful cellular penetration of HIV may be further reduced by any pre-existing micro or macro lesions in the epithelium as well as abrasions upon coital stirring [15], [16]. Thus, in the absence of an established mathematical model that can accurately recapitulate HIV penetration of the squamous epithelium, we chose virion passage through the CVM layer as the time scale to evaluate Ab coverage on virions. Similar assumptions were previously made by the Katz group to model the efficacy of microbicides against HIV [17], [18].

**Table 1:** Parameters and values incorporated into the model.

Category	Parameter	Symbol	Value	Reference(s)
**HIV-1**				
	Radius	r_HIV_	50 nm	[19]
	Diffusivity in semen		Assume same as in CVM	
	Diffusivity in CVM	D_v_	1.27 µm^2^/s i	[12]
	Viral load in semen		8.4×10^5^ copies/ejaculate ii	[47] ^,^ [48]
	Number of Env trimer spikes	N_*_	14+/−7 (s.d.)	[19]
**bnAb (IgG)**				
	Diffusivity in semen		Assume same as in CVM	
	Diffusivity in CVM	D_Ab_	40 µm^2^/s	[11], [32]
	bnAb conc in CVM		Variable	
	bnAb – Env affinity	k_on_, k_off_	Variable; see Table 2	
**Vagina**				
	Surface area of lumen	SA_vagina_	145 cm^2^ iii	[49], [50]
	Volume of luminal CVM	V_CVM_	∼750 uL	[51], [52]
	Thickness of CVM Layer	H_CVM_ iv	50 µm v	
	Volume of semen	V_Semen_	∼3.0 mL	[48]
	Thickness of semen layer	H_Semen_ iv	200 µm v	

i Geometrically averaged D_eff_ for HIV was previously measured to be 0.25 µm^2^/s, but with substantial fraction of viruses exhibiting more rapid mobility. For the current analysis, we used 1.27 µm^2^/s, which represented the top 25^th^ percentile of virus mobility; this is in reasonable agreement with a more recent study of HIV diffusion in genital secretions [53].

ii Estimated based on a median semen volume of 3.0 mL [48], and 2.8×10^5^ HIV-1 RNA copies/mL, which represents the upper limit of HIV-1 RNA copies/mL in seminal plasma from [47]. This is in reasonable agreement with another report by Chakraboty *el al.*, which estimated 5×10^5^ HIV-1 RNA copies/ejaculate, with a maximum of about 2×10^7^ HIV-1 RNA copies/ejaculate [54].

iii The mean surface area of the vagina in the native state was previously estimated to be ∼90 cm^2^ by injection of vinyl polysiloxane casts vaginally. Alternatively, surface area of vaginal lumen may also be inferred by the surface area of erect penis (average ∼200 cm^2^) assuming complete insertion into the vagina. We took the average from the two approaches.

iv In the Materials and Methods section, H_CVM_ is referred to as d and H_CVM_+H_Semen_ = L.

v H_CVM_ estimated by V_CVM_/SA_vagina_; H_Semen_ estimated by V_Semen_/SA_vagina._

The vaginal epithelium is highly folded into collapsed “rugae” coated with a layer of viscoelastic and adhesive cervicovaginal mucus (CVM) gel (Figure 1, top left panel). During coitus, the epithelium becomes stretched and exposed. We thus model the vaginal epithelial surface as the inner surface of a simple cylinder coated with a roughly d = 50 µm thick CVM layer containing different concentrations of elicited or topically dosed bnAb. The thickness of the CVM coating is estimated based on total volumes of mucus that can be collected in the absence of coital stimulation, and assumed to be constant and uniform (see footnote (ii), Table 1; Figure 1). Following ejaculation, seminal fluid is assumed to evenly overlay the CVM layer with a thickness of ∼200 µm, with virions uniformly dispersed within the seminal secretions at a density of 2.8×10^5^ virions/mL (Table 1). Due to rapid diffusivity of protons, semen-mediated neutralization of CVM is assumed to occur instantaneously; we have previously found HIV virions readily diffuse across pH-neutralized CVM, but not acidic native secretions from women with healthy, lactobaccili-dominated vaginal flora [12]. Virions, with 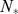 = 14±7 Env trimers (range 4–35) [19], are assumed to maintain native infectivity for the entire duration of the model; thermal degradation based on gp120 shedding (T_1/2_ ∼ 30 hrs) and thermal degradation from RNA polymerase decay (T_1/2_ ∼ 40 hrs) were not incorporated because of the substantial difference in the rate of these processes from the time scale of interest [20]. Because the kinetics of HIV virions penetrating the vaginal epithelium and reaching target cells in the submucosa remain poorly understood, the mobility and number of bound bnAb on each virion is simulated until the virion reaches the vaginal epithelium, or at the end of 2 hrs, whichever comes first. The affinities for different bnAb to purified gp120 as measured by SPR, as well as the corresponding IC_50_ and IC_80_ against the HIV strains from which the purified gp120 are derived, are listed in Table 2. It is important to note that there are substantial variations in the approaches used to measure binding affinities, which range from the use of monomeric gp120 binding to immobilized IgG, to Fab binding to directly immobilized monovalent gp120, to IgG binding to directly immobilized, uncleaved trimers but fitted with a model for monovalent interaction, and finally the binding of uncleaved trimers to captured Fabs, a potentially trivalent interaction, fitted with a monovalent model. None of these approaches would yield the perfect k_on_/k_off_ values for the model here, but in the absence of other reported binding affinity values, we use the currently available literature values as a first estimate. We subsequently included a phase diagram that explores in detail how variations in k_on_/k_off_ might impact our conclusions.

**Table 2:** Binding kinetics and neutralization potencies of bnAb.

Ab	Env strain & type	k_on_ [M^−1^s^−1^]	k_off_ [s^−1^]	IC_50_ [µg/mL]	IC_80_ [µg/mL]	Reference(s)
b12*	YU2 gp120	4.85e4	1.85e-3	2.2	7.8	[10]
b12	JRFL gp120	7.06e4	4.74e-3	0.022	0.075	[10], [55]
b12	JRCSF gp120	1.73e5	4.77e-3	0.096	0.874	[10], [56]
2G12	92UG037.8 gp140	8.4e3	6.0e-3	45.24		[57], [58]
2G12*	HXB2 gp120	1.83e5	1.08e-3	1.01	2.19	[1], [59]
VRC01*	YU2 gp120	1.43e4	5.56e-5	0.126	0.372	[10]
VRC01	YU2 gp140	1.83e4	8.08e-6	0.12	0.372	[60]
VRC01	92UG037.8 gp140	1.6e4	6.4e-5	0.035	0.130	[1]
VRC03*	YU2 gp120	1.33e4	9.74e-4	0.037	0.115	[10]
NIH45-46*	YU2 gp140	4.26e4	2.87e-4	0.05	0.08	[60]
PG9	ZM109 gp120	2.95e4	2.85e-3	0.106	2.64	[1]
PG9	C97ZA012 gp140	1.4e4	2.5e-3	8.20	>25	[57]
PG9*	92UG037.8 gp140	1.9e4	1.0e-3	0.04	0.17	[57]
PG16	C97ZA012 gp140	1.6e4	4.2e-3	2.90	>25	[57]
PG16*	92UG037.8 gp140	2.4e4	1.8e-3	<0.01	0.03	[57]
VRC-CH31	C97ZA012 gp140	9.7e3	1.3e-4	0.18	0.47	[57]
VRC-CH31*	92UG037.8 gp140	8.9e3	4.0e-5	0.04	0.08	[57]

Asterisks indicate k_on_ and IC values that were used in our model to generate Figures 3B, 4 and Figure S2 in File S1.

### Modeling simultaneous diffusion of HIV-1 and Env-binding Ab

We model viruses and bnAb undergoing Brownian motion in CVM/semen mixture, assuming coital stirring motion does not influence the movement of virions into the epithelial layer, due to the viscoelastic nature of CVM. When mucus is sheared between two surfaces, adhesive contacts and entanglements between mucin fibers are drawn apart and a slippage plane forms parallel to the two surfaces, which is reflected by the shear-thinning rheological profile of mucus [4], [21]. Thus, while the viscous drag between the surfaces drops considerably, enabling mucus to function as an effective lubricant, the gel layers adhering to both surfaces remain unstirred even in the presence of vigorous shearing actions. Hence viruses in semen are unlikely to get easily stirred into the mucus layer adhering to the vaginal epithelium.

We model the dynamics of virions and bnAb in two ways: a hybrid stochastic/deterministic system in which we simulate individual virion paths each with unique bnAb binding and unbinding timelines, and a fully deterministic system in which the binding and unbinding rates are expressed in terms of virion and bnAb concentrations. Deterministic models of the virion-bnAb binding kinetics have been previously described by Geonnotti and Katz [18] and more recently by Magnus [22]. The model of Gennotti and Katz also incorporates much of the same biophysical geometry as presented here, while the model developed by Magnus *et al.* [22], [23] provides a rich investigation of the neutralization of virions that results from bnAb binding. When viral concentrations are low, the dynamics are intrinsically stochastic. Our stochastic/deterministic hybrid model yields more detailed information concerning the distribution of possible events and reveals the important role played by constraints to the length and time scales appropriate for *in vivo* dynamics.

In the stochastic/deterministic hybrid model, we describe the movement of an individual virion through the CVM layer by one-dimensional Brownian motion:
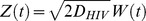
where 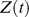 denotes the distance from the epithelial layer of a virion at time 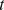, 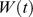 denotes standard Brownian motion, and 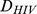 is the viral diffusivity. This diffusion coefficient was assumed to be constant and independent of the evolving number of bound bnAb; the increase in hydrodynamic diameter of Ab-virus complex, even when the virion is completely saturated with Ab, is unlikely to be more than 5–10 nm on a 100 nm virion, and thus assumed to be negligible. At the time of ejaculation, virions are assumed to be uniformly distributed throughout the seminal fluid layer, 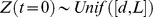, where 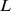 is the distance of the semen-air interface from the epithelium, 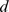 is thickness of the CVM layer, with 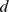 and 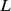 estimated as 50 µm and 250 µm, respectively (see Figure 1). The boundary at 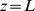 (i.e., the semen-air interface) is considered to be reflecting, while the boundary between the mucus and the epithelial layer 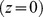 is absorbing.

There are severe computational limitations to direct simulation of the diffusion of 10^12^ IgG molecules with specification of their respective distances from the closest virions. Consequently, we adopted a continuum model to describe the average local concentration of bnAb molecules available to bind HIV virions, an approach used by the Katz group to model the dynamics of microbicide protection against HIV [17], [18]. We assume that bnAb are uniformly distributed throughout the CVM layer (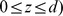 at the time of ejaculation (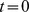). The diffusion of bnAb into the seminal fluid layer is described by the diffusion equation over the region 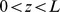:
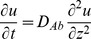
where 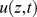 is the local concentration of antibodies, with initial condition 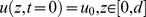 and reflecting boundary conditions at both 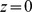 (mucus-epithelia interface) and 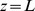 (air-semen interface).

### Kinetics of Ab accumulation on HIV-1

The dynamics of bnAb accumulation on HIV virions depend on (i) bnAb binding affinity to the Env spike (which incorporates the “on rate” k_on_ for binding and “off rate” k_off_ for unbinding), (ii) the local bnAb concentration surrounding the virions (which determines the encounter rate), and (iii) the number of available, bnAb-free Env spikes on the virion. To formulate the equations describing the reaction kinetics (see also [18], [22]), we introduce the notation 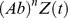 to indicate an HIV virion with 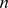 bound bnAb that is located at distance 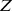 from the epithelial layer at time 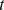. To mimic the observed distribution of 14±7 Env trimers (range 4–35) [19], we select the simulated number of Env trimers 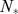 from a Negative Binomial distribution with parameters chosen to yield the observed mean and standard deviation. With the exception of bnAb with one Fab bound per trimer (e.g., PG9), there are 3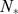 gp120 epitopes available for bnAb binding per Env, and the reaction kinetics can be summarized by the following rates for Ab binding and unbinding between 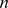 and 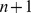 bound bnAb states:
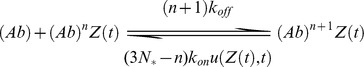



Mathematically, we treat 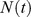
–a time-dependent stochastic process for the number 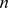 of bnAb bound to a given virion–as a continuous time random walk (CTRW) on the values 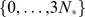 with Markovian transitions because we assume for simplicity that the probability of gaining or losing an antibody depends exclusively on the state of the virion-bnAb system at time 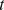. The respective probabilities of gaining an antibody, losing an antibody, or undergoing no change at a location 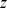 during an infinitesimal time increment of size 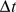 are:
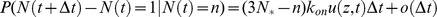


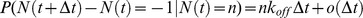


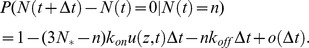



The first equation, for example, asserts that the probability of a binding event occurring in the small time interval Δ*t*, i.e., 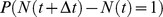, conditional on currently being bound by 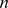 bnAb, i.e., 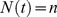, is proportional to the local bnAb concentration 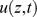 and the total number of available spikes 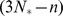. The probability of more than one binding event is higher order in 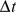 [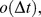
that is, going to zero faster than 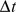 in the limit of small 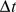] [24]. Because the number of bnAb is large compared to the number of virions, even at relatively low concentration (e.g., 0.01 µg/mL), we ignore local depletion of unbound bnAb that may occur after a binding event. Because the reaction rates are time dependent we implemented a Poisson thinning method [25], which is described in the supplemental text in File S1.

**Figure 2:**
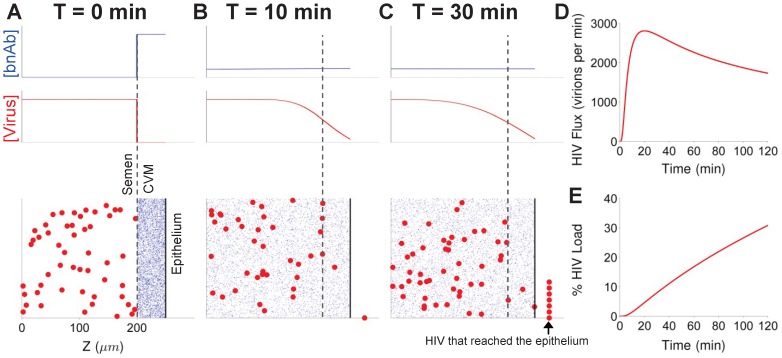
Diffusion of HIV from seminal secretions across CVM to the underlying vaginal epithelium. (A–C) Concentration profile of HIV and broadly neutralizing antibodies (bnAb) in the semen and CVM layers at (A) T = 0 min, (B) T = 10 min, and (C) T = 30 min. (D) Flux of HIV virions arriving at the vaginal epithelium over the first two hours post-ejaculation. 2000 virions correspond to roughly ∼0.25% of the HIV viral load (estimated by RNA copy numbers) in semen. (E) The fraction of total HIV viral load in semen that has penetrated across a 50 µm CVM layer over the first two hours post-ejaculation.

**Figure 3:**
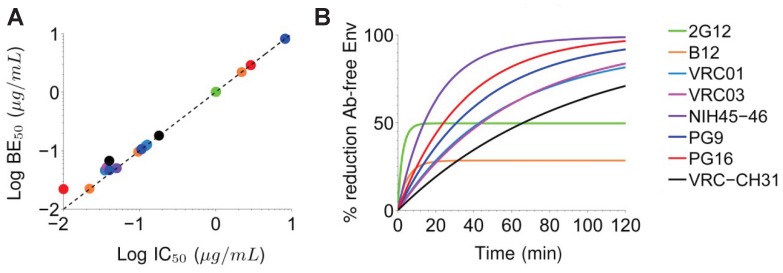
Accumulation of different bnAb on HIV virions over time. (A) Concentration of bnAb necessary to bind to 50% of the Env spikes of HIV (BE_50_) vs. *in vitro* IC_50_ measurements. Dashed line indicates that a 50% reduction in fraction of available Env trimers directly correlates to 50% drop in overall HIV infectivity *in vitro*. (B) Kinetics of bnAb accumulation on HIV virions over the first two hours, as measured by the reduction in fraction of bnAb-free vs. total Env spikes. The k_on_ and k_off_ values for different bnAb in Figure 3A are listed in Table 2; selected ones used in Figure 3B are highlighted in the table.

**Figure 4:**
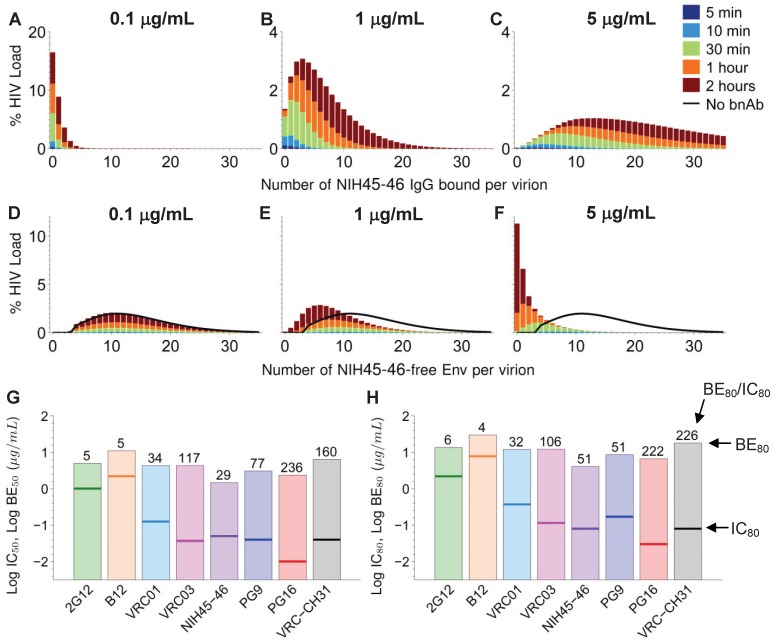
Accumulation of NIH45-46 on HIV virions that have diffused across CVM over the first two hours post-ejaculation. (A–C) Distribution of number of NIH45-46 bound per HIV virion that penetrated CVM, where the initial NIH45-46 concentrations in CVM is (A) 0.1 µg/mL, (B) 1 µg/mL and (C) 5 µg/mL. HIV virions are assumed to have n = 14±7 Env spikes; IC_50_ of NIH45-46 for given k_on_/k_off_ pair (YU2 gp140) is ∼0.050 µg/mL. (D–F) Distribution of number of NIH45-46-free Env spikes on HIV virions that penetrated CVM, where the initial NIH45-46 concentrations in CVM is (A) 0.1 µg/mL, (B) 1 µg/mL and (C) 5 µg/mL. (G–H) Estimated initial concentration of different bnAb in CVM necessary to reduce the average number of bnAb-free Env trimers by (G) 50% (i.e. BE_50_) and (H) 80% (i.e. BE_80_) (indicated by bars), compared to previously reported IC_50_ and IC_80_ values for the respective bnAb (indicated by lines). Listed number in (G) and (H) above each bar represents the ratio of BE_50_ vs. IC_50_ and BE_80_ vs. IC_80_, respectively.

**Figure 5:**
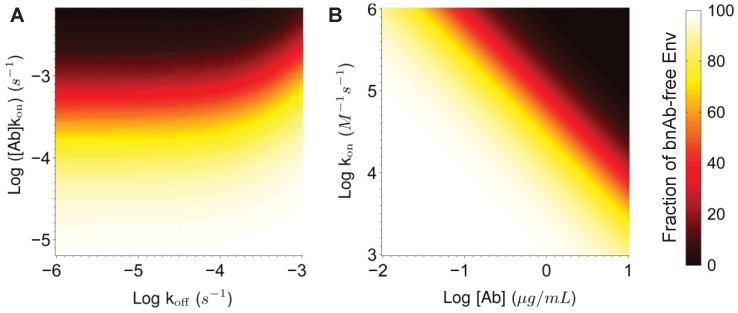
Phase diagrams correlating the kinetic constants (k_on_, k_off_) and Ab concentration necessary to achieve the desired average reduction in the fraction of Ab-free Env spikes for HIV virions that have penetrated CVM during the first 2 hours post-ejaculation. (A) The product of k_on_ and initial bnAb concentration in CVM vs. k_off_. (B) Effects of k_on_ vs. initial bnAb concentration in CVM, assuming k_off_ is 1×10^−4^ s^−1^.

In order to validate our conclusions from the above probabilistic discrete event model, we compare results from a continuum model for both the virus and bnAb populations to binding kinetics modeled via coupled partial differential equations describing the local concentrations of virions with each possible number of bound bnAb. This is valid when both populations are very large. In this formulation, virus concentration is given as a vector 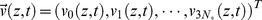, with each component of the vector indicating the concentration profile of virions bound by the given number of bnAb. Instead of a Brownian motion description of individual particle paths, the virus concentration for each vector component was modeled by the diffusion equation 
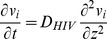
, for 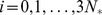. Boundary conditions are absorbing at 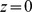 and reflecting at 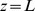. The Forward Time Central Space scheme was used to evolve the diffusion equation. Then the flux for each component at the boundary 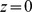 is measured with Fick’s law: 
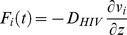
. The Markovian probabilities for gain, loss, and no change in the time increment 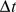 specify transition probabilities in a matrix 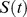, depending on the kinetic rate constants and on the evolving bnAb concentration 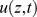, which we use to update the bound populations in a first-order implementation of the form 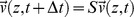. The continuum model agrees extremely well with the Brownian/continuum model for both virus and bnAb populations, and is used to generate Figures 2–5.

It should be noted that a continuum model for bnAb population fails to rigorously account for the propensity for a given bnAb to immediately rebind to a virion after unbinding, either at the same or neighboring gp120 site. To compensate for the resulting overestimate in effective unbinding rates, which is reflected in neutralization statistics IC_50_ and IC_80_ that do not correlate precisely with measured reaction rates k_on_ and k_off_, we adopt an effective unbinding rate for k_off_ as described in the following section.

### bnAb neutralization of HIV

Determining the number of bnAb required to neutralize a given virion remains an active area of research, due to the difficulty in simultaneously distinguishing the number of Ab necessary to neutralize a particular Env spike and the minimum number of Ab-free Env spike necessary for HIV to successfully infect [23]. It was previously proposed that the binding of a single Ab molecule to an Env spike is sufficient to inactivate the infectivity associated with that spike [26]. The minimum number of Ab-free Env spikes, and consequently the number of Env spikes that must be inactivated to neutralize a virion, remain more controversial. Estimates for minimum infectivity ranged from a single Ab-free Env spike [26] to many [27], [28]. For our current model, we assume that each additional Ab binding to a previously unoccupied Env incrementally reduces the likelihood of infection. To validate the hypothesis that neutralization scales approximately with the decrease in the number of Ab-free Env spikes, we performed a steady state analysis of bnAb accumulation on HIV using reported binding affinities, and compared the results to their corresponding IC_50_ values. When considering neutralization studies, the model simplifies significantly because bnAb concentration is assumed to be well-mixed and uniform 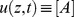 for all 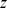 and 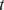. Assuming that each binding site interacts with the bnAb population independently, the distribution of the number of free Env is readily shown to be a Binomial distribution on 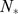 independent trials with a time-dependent success probability, 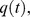 having the form
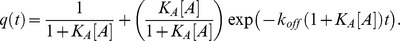
where *K*_A_ = 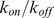. Letting 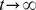, the first term 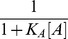 represents the steady-state probability that a given gp120 site is free. The second term reflects that the probability of being in the transient, initial bnAb-free state at time zero decays exponentially with rate 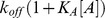. It follows that the fraction of Ab-free gp120 sites in steady state is binomially distributed with 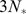 trials and success probability 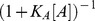. Therefore the steady-state probability that all three gp120 epitopes on a given Env trimeric spike are free of bound Ab is simply 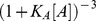.

We found general agreement between IC_50_ values and the predictions of this model. However, the level of agreement was not entirely satisfactory. We believe this is because the model, by adopting unbinding rates measured by SPR techniques in the presence of fluid flow, likely fails to capture the rapid rebinding of bnAb molecules that have recently become unbound, but remain in very close proximity to the binding surface of the virion. This leads to an overestimate of the effective unbinding rate and consequently an underestimate of total bound bnAb. To compensate for this gap – when attempting to emulate the dynamics of known bnAb – we took the IC_50_ values as primary indicators of bnAb performance, and developed a set of “effective k_off_” values that calculate the kinetic rates necessary for the model to produce the observed neutralization data.

Assuming a single bnAb bound to one of the three gp120 sites is sufficient to render that particular Env spike non-infectious [23], [26], [29], the reduction in the mean fraction of Ab-free Env spikes on a HIV virion population is expected to correlate directly with the drop in viral infectivity [30], [31]. This enables direct calculation of an effective k_off_ from experimentally derived IC_50_ and k_on_ values, namely an effective k_off_ (denoted 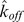) should satisfy 
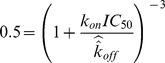
. In other words, at steady state with a concentration of bnAb equal to IC_50_, the combination of 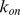
and 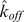 results in 50% of the Env spikes being Ab-free. For a given time scale T, we denote the bnAb concentrations that reduce the average Ab-free trimers across the viral population by 50% and 80% as BE_50_ 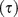 and BE_80_ 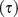 (BE = Bound Env), respectively. Naturally, BE_50_ 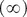 and BE_80_ 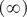 values at steady state calculated from k_on_ and corrected effective k_off_ directly scale with *in vitro* IC_50_ and IC_80_. In virtually all cases, the corrected effective k_off_ was lower than experimentally measured k_off_ (i.e., exhibiting greater affinity to Env). Our goal in assessing how *in vitro* performance compares to that *in vivo* essentially involves the calculation of BE_50_ 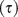 and BE_80_ 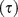 where 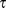 is the random time it takes for a virion to traverse the CVM layer. We observe strong agreement between these calculated BE_50_ 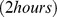 values to the observed IC_50_ (Figure 3A). While the introduction of an effective k_off_ helps bring the predictions of the kinetic model in line with *in vitro* neutralization studies, we emphasize that this does not affect our conclusion of the relative importance of k_on_ vs. k_off_ *in vivo.* This fact is captured in the heat map representation of neutralization given in Figure 5: changes in k_off_ by log-decades yield only marginal improvement in predicted *in vivo* protection. Indeed, this is observed even when we remove the possibility of unbinding and set k_off_ to zero.

## Results

### HIV-1 can quickly traverse CVM layers

IgG, the predominant Ab found in CVM rather than IgA [1], [2], diffuses largely unhindered through mid-cycle human cervical mucus [11], [32]. Owing to this high diffusivity, IgG in CVM readily enters the semen layer, and achieves essentially uniform distribution across the CVM/semen mixture within minutes (Figure 2). In contrast, although HIV virions are readily mobile in pH-neutralized human CVM, their effective diffusivity is over 30-fold slower than that of IgG molecules [12]; thus, the distribution of HIV across semen/CVM mixture remains non-uniform even after 2 hrs, with the majority of the virions (∼70%) still retained in the seminal secretions. Nevertheless, the first virions can reach the vaginal epithelium very quickly, and it takes only ∼10 and ∼20 mins post-ejaculation before ∼1% and ∼5% of the total HIV virions in semen (corresponding to an average of 8.4×10^3^ and 4.2×10^4^ RNA copies, assuming a median of 8.4×10^5^ RNA copies per ejaculate) can reach the vaginal epithelium, respectively. The peak HIV flux (rate of virions reaching the epithelium) occurs roughly 20 mins post-ejaculation, and within 2 hrs nearly 30% of the initial HIV load has reached the vaginal epithelium. These results highlight the limited time window for bnAb in CVM to neutralize HIV virions in the vaginal lumen.

### Rates of bnAb accumulation on HIV virions are slower than generally thought

We find that many of the recently discovered bnAb require more than 1–2 hrs to reach the steady state number of bound bnAb per virion (Figure 3B). Even at a bnAb concentration of 1 µg/mL, which is roughly 10- to 20-fold higher than the IC_50_ and IC_80_ for many bnAb-HIV strain pairs (with the exceptions of b12 and 2G12 against the YU-2 and HXB2 strains, respectively), steady state virion binding is not observed for most bnAb after the typical 1 hr incubation period. These results confirm recent data by Ruprecht *et al.*, which showed neutralization potencies of bnAb steadily increase with increasing Ab-virus incubation period from 1 hr to 20 hrs [33]. Together, these results underscore the slow binding kinetics of bnAb to HIV despite their exceptional neutralization potencies as measured by standard pre-clinical neutralizing antibody assays. Because our model approximates the drop in infectivity to the decrease in the fraction rather than absolute number of bnAb-free Env spikes, the neutralization kinetics of different bnAb is only weakly dependent on the actual number of Env spikes on native HIV virions.

### Effective neutralization in CVM requires markedly higher bnAb concentrations than *in vitro* estimates

To evaluate the competing time scales of binding and diffusion *in vivo*, we combine the above virus diffusion rates and Ab-binding kinetics to estimate the number of bound bnAb, residual Ab-free Env proteins and relative infectivity for each HIV virion that successfully diffuses across the CVM layer and reaches the vaginal epithelium (Figure 4). We use NIH45-46, one of the most potent bnAb reported to date [8] and among the fastest to achieve equilibrium binding among the recently discovered bnAb in our model, as the reference. The majority of viruses that reached the vaginal epithelium have either zero or one NIH45-46 bound to them at initial CVM concentration of 0.1 µg/mL (Figure 4A & 4D; Movie S1; IC_50_ = 0.05 µg/mL for YU2 strain of HIV used for measuring affinity by SPR), suggesting NIH45-46 is unlikely to protect against vaginal transmission of YU2 strain virions *in vivo* at that particular concentration. Raising the NIH45-46 concentration in CVM to 1 µg/mL only modestly increases the number of HIV-bound NIH45-46 (Figure 4B, 4E; Movie S2). A significant increase in bnAb coverage, and corresponding drop in bnAb-free Env, is only achieved at bnAb levels approaching 5 µg/mL (Figure 4C, 4F; Movie S3). Indeed, our estimated BE_50_ and BE_80_ values for NIH45-46 against virions that penetrate CVM are 30- and 50- fold greater than the *in vitro* IC_50_ and IC_80_ values (all discussion of BE_50_ and BE_80_ values from here on refers to bnAb concentrations necessary to reduce the fraction of Ab-free Env on virions that penetrate CVM within 2 hrs by 50% and 80%). Much of the decrease in mean residual Ab-free Env is observed with virions that took relatively longer times (i.e., >30 mins) to penetrate the CVM layer. Even at 5 µg/mL initial Ab concentration in CVM, the fraction of NIH45-46-free Env sites is not substantially reduced on virions that penetrate CVM within the first 30 minutes post-ejaculation. Similar results are obtained even under the extreme modeling assumption of fully suppressed Ab unbinding; with k_off_ = 0, the BE_50_ and BE_80_ for NIH45-46 are 1.3 and 3.8 µg/mL, respectively, which are still roughly 25- and 50-fold greater than *in vitro* IC_50_ (0.05 µg/mL) and IC_80_ (0.08 µg/mL) values. This suggests that improvement in k_off_ alone is not likely to improve protection *in vivo*.

We next estimate the bnAb accumulation kinetics for other bnAb. For most of the recently discovered bnAb investigated with our model, BE_50_ and BE_80_ values on virions that successfully penetrate the CVM layer are at least 30- to 250-fold higher than reported *in vitro* IC_50_ values. Our result is in good agreement with studies from numerous groups that showed protection by passive transfer of bnAb in macaques required concentrations substantially greater than *in vitro* estimates [34]–[37]. The discrepancy between the *in vitro* IC_50_ and our predicted BE_50_ values (or IC_80_ vs. BE_80_) can be reduced by increasing k_on_ (Figure 4G & 4H; Figure S1 in File S1). Indeed, the two Ab with the highest k_on_ in our current analysis, 2G12 and b12, exhibit BE_50_ only 5-fold higher than their IC_50_ estimates, despite their markedly weaker neutralizing potencies. Unlike many of the bnAb, 2G12 targets mannose residues that are relatively accessible on the gp120 “glycan shield” [38], which likely accounts for its rapid binding kinetics. Despite having one of the fastest unbinding rates, 2G12 has been established as one of the most potent bnAb under *in vivo* conditions [39]. For example, in a study from the Burton group [34], three out of five macaques infused with 2G12 were completely protected from infection, with one exhibiting delayed viral appearance and diminished replication; in contrast, all 4 control macaques became infected. The 90% neutralization titers (IC_90_) for serum 2G12 in these animals was 1∶1, suggesting 2G12 can offer substantial protection at relatively low serum neutralizing titers [34].

### The impact of k_on_ on neutralization kinetics is insensitive to the choice of infectivity threshold model

As discussed in the Materials and Methods section, there are a number of infectivity models in the literature. To date, no experimental setup has yielded sufficient resolution to identify the primary mechanism among these various candidates. In this paper, we assumed infectivity scales with the fraction of Ab-free Env. To ensure that our overall conclusions do not rest on this assumption, we compared our results to the outcome of numerical experiments using a minimum threshold model. In this alternate model, in order for a virion to be infectious at all, we require that there must be at least 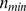 Ab-free trimers on the virion’s surface. The infectivity function is then incrementally increased for each additional Ab-free trimer with relative infectivity given by 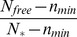.

Figure S2 in File S1 shows the resulting modifications to 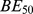 and 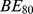 for the various Ab utilized in our simulations, as well as the comparison to the respective published 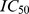 and 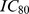 values. All 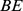 values move closer to 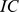 values for larger 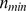, but the large discrepancy between 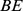 and 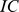 values (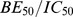 and 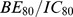) is significant for all Ab other than 2G12 and b12, the two bnAb that possess the greatest 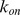 values in our list of bnAb-HIV strain pairs. Furthermore, the ratio between the 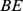 and 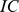 values for various Ab is similar regardless of 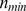 (Figure S3 in File S1), suggesting that the choice of infectivity model does not significantly affect the conclusions of this study.

### Effectiveness of HIV neutralization in CVM is likely limited by k_on_ rather than k_off_

In the above analysis, bnAb exhibiting the lowest BE_50_ and BE_80_ are also those with rapid k_on_, since these Ab can most quickly engage an HIV virion and reduce its infectivity prior to the virion reaching target cells. We thus seek to further analyze the antibody-antigen affinity characteristics that would most likely effectively neutralize HIV virions during male-to-female vaginal transmission. We find that Ab concentration and k_on_ most directly influence the reduction in mean number of Ab-free Env proteins on HIV that reached the vaginal epithelium, whereas k_off_ has a relatively minor effect under these conditions. As long as effective k_off_ is less than 10^−4^ s^−1^, BE_80_ can be achieved when the product of k_on_ and Ab concentration exceeds 1.3×10^−3^ s^−1^. Assuming that sustainable bnAb levels elicited by vaccination are unlikely to exceed ∼1% of the total vaginal IgG levels in CVM (mean: ∼540±110 µg/mL), a BE_80_ equivalent to this concentration must exhibit a k_on_ in excess of 3.5×10^4^ M^−1^ s^−1^. This k_on_ requirement may be reduced if higher bnAb concentrations can be achieved by topical prophylaxis.

## Discussion

Although often under-appreciated, CVM represents the first line of defense against sexually transmitted infections in the female reproductive tract. In addition to minimizing trauma to the vaginal epithelium upon coital stirring, the presence of the CVM layer also prevents virions in semen from immediately contacting the vaginal epithelium upon ejaculation, and directly reduces the virion flux and total viral load in semen that can reach target cells over time. Unfortunately, since HIV is generally not slowed substantially in CVM, there is only a very limited window of opportunity during which secreted or topically delivered Ab can bind to and neutralize HIV before the virion encounters target cells. Based on published measurements of bnAb affinity to gp120/gp140 trimers, our model predicts that many monoclonal bnAb, at IC_50_ and IC_80_ levels measured *in vitro*, are likely unable to comparably neutralize most HIV strains within the time scale of virion diffusion through the CVM layer. Thus, despite the orders of magnitude improvements in *in vitro* neutralization potencies that extend IC_50_ and IC_80_ for many HIV strains to the ng/mL levels, bnAb levels in excess of 5–10 µg/mL in CVM prior to coitus may be necessary to reduce rates of transmission of diverse strains of HIV by 50–80%, especially against the virion outliers that traverse the CVM layer most rapidly (Figure 4G & 4H). While the failure of bnAb-based pre-exposure prophylaxis is frequently attributed to poor extravasation of systemic IgG into genital secretions or non-uniform distribution of topically delivered bnAb in the vaginal lumen, our model introduces a third mechanism – inadequate neutralization kinetics, especially against rapidly diffusing virions – as another potentially important challenge to ensure sterilizing vaginal immunity against HIV.

The need for relatively high levels of bnAb in CVM is likely attributed in part to a striking dilution effect due to rapid diffusive mixing of the Ab in semen and CVM: effective bnAb levels in CVM are reduced at least ∼5-fold relative to native CVM levels within minutes of ejaculation. While increasing initial bnAb levels in CVM is the most obvious and direct approach to improve the rates of bnAb accumulation on HIV, our model suggests another potential tactic to enhancing vaginal immunity against HIV is to focus on bnAb that quickly bind HIV virions, and not necessarily just Ab with the highest affinity. A number of reports have correlated the kinetic rates and affinity of different Ab to Env proteins to their neutralization potency against HIV; low k_off_ and K_D,_ rather than high k_on_, is often thought to be essential to effective neutralization of the virions [40]. However, by assessing the neutralization potency of Ab after an initial incubation period of typically at least one hour with the virions prior to exposure to target cells, these *in vitro* studies likely partially masked the importance of k_on_ to HIV neutralization *in vivo*. Thus, it is not surprising that a low k_off_, which influences the rates with which Ab-bound Env becomes free of Ab as well as the steady state fraction of Ab-free Env proteins, correlated well with the most potent Ab. Since typical *in vitro* screening does not distinguish Ab molecules with rapid k_on_, the potential need for rapid neutralization kinetics has received little attention amidst the current search for monoclonal bnAb capable of neutralizing diverse HIV strains. The potential importance of k_on_ was previously raised in a study by Steckbeck *et al.*, who observed a significant correlation between association rates but not the affinity of Ab binding to SIV/17E-CL and SIVmac239 envelope proteins and the neutralization sensitivities of the corresponding virus strains [41]. Ab association rates, rather than dissociation rates, also appear to play a predominant role in the neutralization of respiratory syncytial viruses (RSV), where palivizumab variants with greater association rates conferred greater neutralization potency [42]. Ab molecules with rapid k_on_ may be naturally selected during the human antibody response to HIV, whereby somatic mutations lead to polyreactive Ab capable of bivalent heteroligation between a high-affinity site on the Env protein and a second low-affinity site on another molecular structure on HIV. Due to the paucity of Env proteins on HIV, these polyreactive Ab can associate on the HIV surface substantially more quickly than non-polyreactive, homotypic bivalent binding Ab typical of many bnAb. In a recent study, nearly 75% of 134 monoclonal anti-gp140 Ab cloned from 6 patients with high titers of neutralizing Ab are polyreactive [43].

The binding affinity for different bnAb to HIV is typically measured on gp120/gp140 trimers purified from a single strain of HIV, such as YU2 or JRFL. These strains are generally considered average to moderately easy to neutralize (e.g., VRC01 IC_50_∶0.126 µg/mL for YU2, 0.031 µg/mL for JRFL) relative to other transmitted HIV strains (geometric mean IC_50_ for VRC01∶0.34 µg/mL excluding strains that require >50 µg/mL to neutralize). Thus, the bnAb binding affinities used in our model are likely representative or potentially even higher than actual affinities to other HIV strains. While the *in vitro* neutralization potency of bnAb against a large panel of HIV strains can be rapidly assessed, no such capability currently exists to measure the binding affinity for different bnAb against diverse HIV strains. We are thus unable to evaluate the effective mucosal neutralization potency for different bnAb against diverse HIV strains in our current model. Nevertheless, we expect HIV strains that neutralize *in vitro* more readily than YU2/JRFL will also be more readily neutralized by the various bnAb at equal or lower CVM concentrations. Correspondingly, HIV strains that are already difficult to neutralize *in vitro* will likely require even higher bnAb concentration in CVM before possible neutralization.

Although our results suggest that bnAb concentrations in marked excess of *in vitro* IC_50_ and IC_80_ must be present in CVM to block vaginal transmission, there is increasing evidence that effective mucosal protection may be achieved with Ab at sub-neutralizing doses or with non-neutralizing Ab. For example, comparable protection was found with two IgGs that exhibited ∼10- to more than 100-fold difference in neutralization potency [34], and the vaccine regimen in the recent Thai RV144 trial enabled ∼60% protection of vaccinated subjects in Year 1 despite inducing a poor neutralizing Ab response [44]. This conundrum might be explained by low affinity crosslinks between IgG and mucins that lead to polyvalent, high avidity immobilization of HIV virions, a potential mechanism of mucosal immunity that remains largely unexplored to date. The diffusion of IgG molecules (diameter ∼ 10 nm) is only slightly retarded in human mucus (pores ∼ 340±70 nm [45]) compared to buffer, indicative of low-affinity, transient crosslinks with the mucus gel [11]. As IgG molecules accumulate on virions such as HIV, the array of bound Ab may form a sufficient number of transient low-affinity bonds to mucins at any given time to effectively trap (immobilize) the virion in the mucus gel. As few as several Ab bound per virion may generate sufficient affinity to mucins to markedly reduce the flux of virions reaching target cells, thereby prolonging the time window for more complete neutralization of fast moving HIV virions likely responsible for infections. We have recently found IgG to mediate effective trapping of Herpes Simplex Virus at sub-neutralizing IgG doses [46], and we are actively investigating whether bnAb may help trap HIV virions in mucus. Nevertheless, because the precise dynamics with which virion-bound Ab may slow virion diffusion are yet to be determined, we did not incorporate this potential mechanism of vaginal immunity by bnAb in our current model. Such trapping of HIV virions in mucus prior to their diffusion to target cells is critically dependent upon achieving maximal virion-bound Ab within a short time window; thus, it is likely that mucosal Ab that efficiently leverage this protective mechanism would also exhibit rapid k_on_.

The thickness of CVM directly influences the time available for bnAb to bind to virions before the virions can penetrate CVM. When the CVM thickness is <50 µm, substantially greater levels of bnAb must be present in native secretions to achieve BE_50_ and BE_80_, whereas lower levels of bnAb are needed with greater CVM thickness. Unfortunately, the precise thickness of the CVM layer remains poorly understood compared to those at other mucosal surfaces such as the lung airways and the eye, due to a series of compounding factors. For example, the volume of genital secretions coating the vaginal epithelium can vary substantially throughout the menstrual cycle, with maximum volume typically occurring during mid-cycle. Genital secretions often decrease with age due to reduction in estrogen levels, and there may be local heterogeneities with little to no mucus present on particular regions of the vaginal epithelium. The volume of vaginal secretions is also influenced by coital stimulation. While increased mucus secretion during coitus or mid-cycle is generally thought to provide lubrication to minimize physical trauma to the epithelial layer (e.g., microabrasions) and consequently decreased risks of infection, it may also represent an evolutionary mechanism to enhance the diffusional and immunological barrier against sexually transmitted pathogens.

Clearance of semen from the female reproductive tract is not incorporated into our current model, largely because of a lack of literature documenting the rates and hydrodynamics of seminal fluid clearance. Nevertheless, assuming there is no gap in the mucus secretions coating the vagina, and that infectious virions had not already reached the vaginal epithelium prior to ejaculation, inducing rapid semen elimination should directly reduce the flux of virions reaching target cells and consequently the rate of male-to-female transmission of cell-free HIV virions. As shown in Figure 2, the first virions are unlikely to diffuse across the CVM until a few minutes post-ejaculation, implying a potentially critical time window for eliminating infectious virions via semen clearance. While this practice clearly should not replace any of the currently available methods for protection against sexually transmitted infections, it adds to the list of behavioral and/or readily adoptable approaches that may reduce HIV or other sexually transmitted infections, which includes reducing the number of sexual partners, increasing condom use, and circumcision.

Our current model is a first step towards an improved quantitative understanding of the dynamics with which HIV establishes infection in the female reproductive tract, and serves as a foundation to incorporate additional antibody-effector functions (e.g., ADCC, complement). Nevertheless, many of the intricacies revealed by our analysis, such as the physiologically relevant timescales for Ab accumulation on virions, provide quantitative insights into strategies to improve humoral immune responses, and should be broadly generalizable to understanding the kinetics of other viral infections at mucosal surfaces. We expect additional iterations and future improvements to our model will provide predictive insights into the Ab doses needed for ensuring protective vaginal immunity against HIV and other sexually transmitted infections.

## Supporting Information

File S1 **Supplemental methods, supporting figures, and supporting figure legends.**
(PDF)

File S2 **MATLAB codes for simulation.**
(ZIP)

Movie S1 **Diffusion of HIV across cervicovaginal mucus with initial NIH45-46 concentration of 0.1 µg/mL, from 0–120 mins post-ejaculation.**
(AVI)

Movie S2 **Diffusion of HIV across cervicovaginal mucus with initial NIH45-46 concentration of 1 µg/mL, from 0–120 mins post-ejaculation.**
(AVI)

Movie S3 **Diffusion of HIV across cervicovaginal mucus with initial NIH45-46 concentration of 5 µg/mL, from 0–120 mins post-ejaculation.**
(AVI)
