# Supplementary material for: Modeling Neutralization Kinetics of HIV by Broadly Neutralizing Monoclonal Antibodies in Genital Secretions Coating the Cervicovaginal Mucosa
Source: PLoS One. 2014 Jun 26;9(6):e100598. doi: 10.1371/journal.pone.0100598 (PMC4072659; doi:10.1371/journal.pone.0100598)
Supplement: File S1 — Supplemental methods, supporting figures, and supporting figure legends. (PDF) [file pone.0100598.s001.pdf]

Supplemental Information

Simulation of non-homogeneous Poisson processes.

If the rates of Ab accumulation on virions are constant over time, then the waiting time between events would be exponentially distributed. That is to say, if we define $\tau(t_0)$ to be the time of the earliest reaction after a time t_0, then $P(\tau(t_0) > t) = e^{-r(t-t_0)}$ with r as the reaction rate. However, because the binding rates depend on the dynamic, time-dependent location of the virions and the bnAb concentration profile, the formula for the probability distribution of the waiting time between binding/unbinding events generalizes to [39, 40]:

$$P(\tau(t_0) > t \mid N(t_0) = n, \{Z(s)\}_{s>t_0}) = \exp[-\int_{t_0}^{t_0+t}(\lambda_n(s) + \mu_n)ds],$$

where the instantaneous binding rate is denoted $\lambda_n(s) = (3N_* - n)k_{on}u(Z(s), s)$, and the unbinding rate is $\mu_n = nk_{off}$. This distribution describes the time until the next binding/unbinding event, but not whether the event is binding or unbinding of Ab from the virion. Since we assume this is a Markov process, given that a reaction event occurred at time $t_0 + \tau(t_0)$, the probability that the event is a binding or unbinding event can be approximated by the relative magnitudes of the instantaneous propensities to bind or unbind at time t_0. Based on the formula above, the probability that an unbinding event took place is $\mu_n/[\mu_n + \lambda_n(t_0)]$, whereas the probability that a binding event took place is $1 - \mu_n/[\mu_n + \lambda_n(t_0)]$.

There are multiple methods for simulating reaction kinetics with rates that are not constant in time. In order to reduce computational complexity, we implement a Poisson thinning method [39, 40]. Conceptually, the method consists of two steps. First, the method simulates binding/unbinding events at a constant rate corresponding to the maximal rate over the simulated time interval, generating a series of "candidate events" (termed as such because the series includes too many events relative to the actual time-dependent process). Second, we screen through the candidate events one by one and reject them according to a probabilistic choice method that results in statistically accurate simulations.

To compute the maximal reaction rates, we first calculate the complete time evolution of the bnAb concentration $u(z, t)$, $z \in [0, L], t \in [0, T]$ as well as the Brownian virion paths over the time interval $t \in [0, T]$. The number N_* of Env trimer spikes is drawn from a negative binomial distribution with mean 14 and standard deviation 7 [25]. The duration between binding/unbinding events $\{\tau_0, \tau_1, \ldots, \tau_k\}$ is then generated sequentially for each virion, as follows: given a reaction time τ_i and the number of bound bnAb at that time, $N(\tau_i)$, we calculate the next reaction time by first computing an upper bound for the reaction rates over the remaining simulation time, $\rho = \max_{\{t\in(\tau_i,T)\}}\{\lambda_{N(\tau_i)}(t) + \mu_{N(\tau_i)}\}$. As described above, a sequence of candidate Poisson event times $\{T_1, \ldots, T_m\}$ were then

generated at rate ρ over the interval $[\tau_i, T]$, each of which is accepted with probability $(\lambda_{N(t_0)}(T_i) + \mu_{N(t_0)})/\rho$. We take the minimum of the accepted candidate times and assign this to be the reaction time τ_{i+1}. The event is treated as an unbinding event with probability $\frac{\mu_{N(t_0)}}{\lambda_{N(t_0)}(\tau_{i+1})+\mu_{N(t_0)}}$ (otherwise, it is a binding event). This process is repeated until no accepted event times remain in the time interval.

Supplemental Figures Legend

Figure S1. Ratio of **(A)** IC_{50}/BE_{50} and **(B)** IC_{80}/BE_{80} vs. k_{on} for different bnAb.

Figure S2. Estimated initial concentration of different bnAb in CVM necessary to reduce the average number of bnAb-free Env trimers by 50% (i.e. BE_{50}) and 80% (i.e. BE_{80}) (indicated by bars), compared to reported IC_{50} and IC_{80} values for the respective bnAb (indicated by lines). The BE estimates are based on a threshold model assuming n_{min} of 5 and 8 bnAb-free Env for virus to be infectious. Listed number above each bar represents the ratio of BE_{50} vs. IC_{50} and BE_{80} vs. IC_{80}, respectively.

Figure S3. Ratio of **(A)** IC_{50}/BE_{50} and **(B)** IC_{80}/BE_{80} vs. k_{on} for different bnAb assuming an incremental model (n_{min} = 0) or a threshold model with n_{min} of 5 and 8.

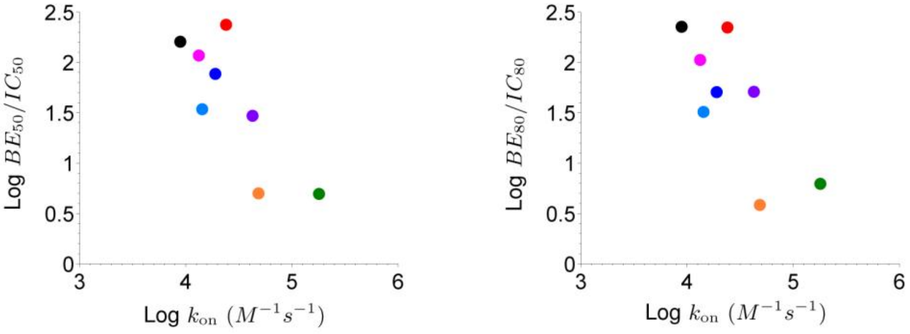

Figure S1

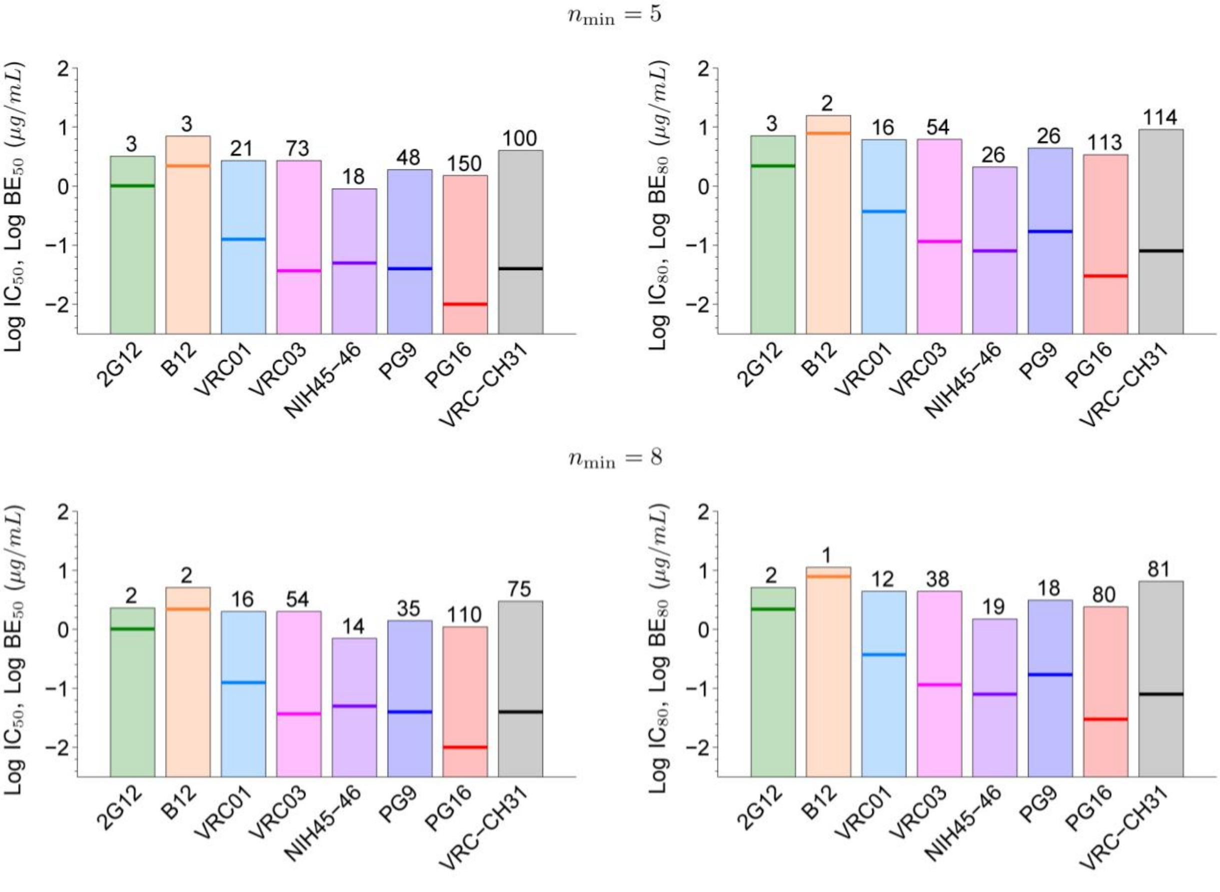

Figure S2

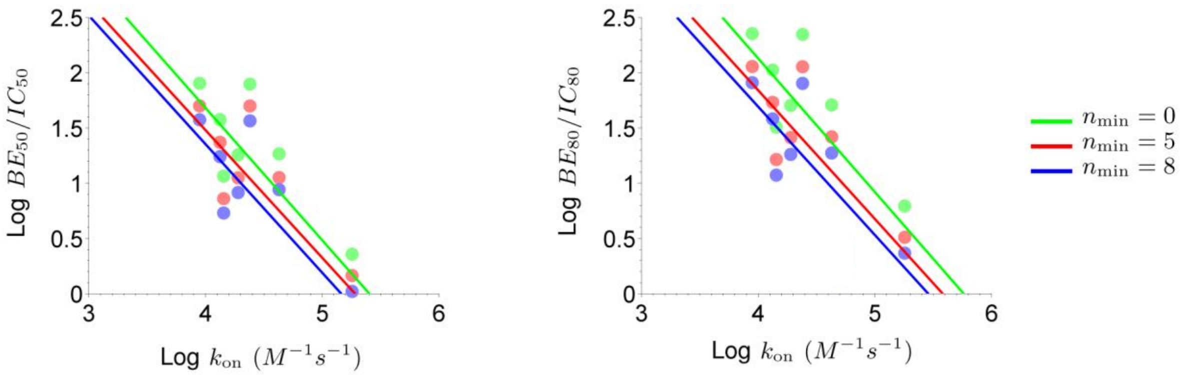

Log BE_{50}/IC_{50}
Log k_{on} $(M^{-1}s^{-1})$
Log BE_{80}/IC_{80}
Log k_{on} $(M^{-1}s^{-1})$
$n_{\min}=0$
$n_{\min}=5$
$n_{\min}=8$

Figure S3
